# Theoretical Study of Light-Induced Crosslinking Reaction Between Pyrimidine DNA Bases and Aromatic Amino Acids

**DOI:** 10.3389/fbioe.2021.806415

**Published:** 2022-01-17

**Authors:** Attila Bende, Alex-Adrian Farcaş, Valer Toşa

**Affiliations:** ^1^ Molecular and Biomolecular Physics Department, National Institute for Research and Development of Isotopic and Molecular Technologies, Cluj-Napoca, Romania; ^2^ Faculty of Physics, Babeş-Bolyai University, Cluj-Napoca, Romania

**Keywords:** DNA–protein crosslinking, cycloaddition reaction, time-dependent density-functional theory, conical intersection, excited-state deactivation pathway, uracil, benzene, phenol

## Abstract

Low-lying electronic excited states and their relaxation pathways as well as energetics of the crosslinking reaction between uracil as a model system for pyrimidine-type building blocks of DNA and RNA and benzene as a model system for aromatic groups of tyrosine (Tyr) and phenylalanine (Phe) amino acids have been studied in the framework of density functional theory. The equilibrium geometries of the ground and electronic excited states as well as the crossing points between the potential energy surfaces of the uracil–benzene complex were computed. Based on these results, different relaxation pathways of the electronic excited states that lead to either back to the initial geometry configuration or the dimerization between the six-membered rings of the uracil–benzene complex have been identified, and the energetic conditions for their occurrence are discussed. It can be concluded that the DNA–protein crosslinking reaction can be induced by the external electromagnetic field via the dimerization reaction between the six-membered rings of the uracil–benzene pair at the electronic excited-state level of the complex. In the case of the uracil–phenol complex, the configuration of the cyclic adduct (dimerized) conformation is less likely to be formed.

## 1 Introduction

The DNA of a living cell is continuously exposed to external influences, which can induce various forms of DNA damage. When this, e.g., appears as a covalent bond of proteins with a DNA strand, through either DNA bases or the sugar–phosphate chain, this phenomenon is called DNA–protein crosslinking (DPC). This is one of the most deleterious forms of DNA damage, which can disturb or even stop the proper cell transcription and replication. To maintain genomic stability, cells must continually repair such damage in a timely manner (Lindahl, 1993; Stingele et al., 2017). DPCs can be induced by exposure to various physical and chemical agents including ionizing radiation, UV light, transition metal ions, environmental contaminants, and common anticancer drugs (Connelly and Leach, 2004; Barker et al., 2005; Ide et al., 2011; Tretyakova et al., 2015).

Electromagnetic radiation such as UV- or visible-light can, through several processes, interact with biological matter and can cause either desired or unwanted biological (Bryant and Frigaard, 2006; Schreier et al., 2007) or chemical changes (Fingerhut et al., 2012; Zhou et al., 2019). DPCs were first recognized as a distinct lesion in UV light-irradiated bacteria by Smith (Smith, 1962) and by Alexander and Moroson (Alexander and Moroson, 1962). Biological changes caused by UV radiation have long been considered as harmful as in cases caused by chemical agents, but they were recognized also as the benefit of the phenomenon. Thus, UV crosslinking may be used to selectively label DNA-binding proteins based on their specific interaction with a DNA recognition site (Stützer et al., 2020), and it has been successfully applied to the study of protein–DNA interactions (Welsh and Cantor, 1984; Dimitrov and Moss, 2001). Furthermore, the UV-induced crosslinking and immunoprecipitation (CLIP) technique is used to study dynamical ribonucleoprotein (RNP) assemblies for identifying the sites bound by a specific RNA-binding protein on endogenous RNAs (Spitzer et al., 2014; Hafner et al., 2021).

A simple model system to explore the possibility of light-induced crosslinking reaction between the pyrimidine bases and the side chains of the aromatic amino acids was experimentally investigated by Sun and Fecko (Sun et al., 2006; Fecko et al., 2007). Since the yield of the photoreaction between the pyrimidine bases and the aromatic amino acids is quite low (Shaw et al., 1992; Sun et al., 2006), a short linker of –CH_2_– group to mimic the proximity and orientation in DNA–protein complexes was applied between the uracil and benzene fragments. Accordingly, for the final photocyclization of the benzyluracil system, a complex, six-step reaction scheme including electron and proton transfers as well as radical ion pair formation was proposed. Given that these photochemical reactions are very difficult to follow, theoretical modeling can reveal many small details in addition to a global understanding of the processes and thus explain intermediate steps that we cannot observe with our experimental tools (Kleinermanns et al., 2013; González et al., 2016). Accordingly, the model system of benzyluracil was also intensively investigated considering different theoretical frameworks, and several photoreaction mechanisms were proposed (Micciarelli et al., 2013; Micciarelli et al., 2014; Bende and Toşa, 2015; Micciarelli et al., 2017; Valadan et al., 2019; Zhao et al., 2021).

Although the inclusion of the –CH_2_– bridge can ensure the proximity of the two fragments, it also determines the outcome of a possible photochemical reaction. In contrast, a simpler photochemical reaction can also take place between two nearby aromatic systems. This phenomenon is known in the literature as thymine dimerization (Boggio-Pasqua et al., 2007; Schreier et al., 2007; Law et al., 2008; Kancheva and Delchev, 2016; Mendieta-Moreno et al., 2016; Zhao et al., 2021). This kind of reaction mechanism does not include any intermediate steps of charge or proton transfer as well as of radical ion pair formation, just a formation of a cyclic adduct (CA) between unsaturated fragments of two molecules.

Our goal is to map using theoretical investigation the possible photochemical processes that may finally occur to light-induced crosslinking reactions between DNA bases and aromatic amino acids.

## 2 Materials and Methods

All equilibrium geometry optimizations and conical intersection (CI) searches were made in the framework of the density functional theory (DFT) considering the ωB97X exchange-correlation (XC) functional (Chai and Head-Gordon, 2008) combined with the D3-type empirical dispersion correction scheme (Grimme et al., 2010; Lin et al., 2013) and applying the minimally augmented (Zheng et al., 2011) ma-def2-TZVPP triple-ζ basis set of the Karlsruhe group (Weigend and Ahlrichs, 2005) as implemented in the Orca program suite (Neese, 2012; Neese, 2018). The electronically excited-state calculations were computed using the time-dependent version of the same DFT framework considering the Tamm–Dancoff approximation (TDA) (Hirata and Head-Gordon, 1999). The RIJCOSX approximation (Neese et al., 2009) designed to accelerate the Hartree–Fock and hybrid DFT calculations were considered together with the Def2/J (Weigend, 2006) auxiliary basis set for the Coulomb fitting and def2-TZVPP/C (Hellweg et al., 2007) auxiliary basis set for correlation fitting in the case of TDDFT calculations. The Nudged Elastic Band (NEB) method (Henkelman and Jónsson, 2000; Ásgeirsson et al., 2021) is used to locate the transition state (TS) geometry and to find the minimum energy path (MEP) connecting the minims of different equilibrium geometries on the potential energy surface. The solvent environment of water was taken into account through the conductor-like polarizable continuum (CPCM) model (Barone and Cossi, 1998) and explicitly included two water molecules close to the two carboxyl groups of the uracil molecule. The interaction energies in different molecular dimers were calculated using the pair natural orbital-based local coupled-cluster method (DLPNO-CCSD(T)) (Hansen et al., 2011; Liakos et al., 2011; Riplinger et al., 2013). Based on the localization of the occupied pair natural orbitals, the total energy (reference energy + correlation) can be obtained as a sum of different intra- and inter-fragment contributions. The inter-fragment interaction energy can be written as follows:

$$\Delta E=\;\delta E_{el.-prep}^{ref.}+E_{elstat.}^{ref.}+E_{exch.}^{ref.}+\delta E_{nonDisp}^{C-CCSD}+E_{Disp}^{C-CCSD}+\delta E_{int.}^{C-\left(T\right)}$$ (1)

where $\delta E_{el.-prep}^{ref.}$ means the electronic preparation (or intra-fragment reference) energy and describes how much energy is necessary to bring the fragments into the electronic structure that is optimal for interaction, $E_{elstat.}^{ref.}$ , and $E_{exch.}^{ref.}$ are the inter-fragment electrostatic and exchange contributions, $\delta E_{nonDisp}^{C-CCSD}$ and $E_{Disp}^{C-CCSD}$ are the non-dispersive and dispersive parts of the correlation energy at the CCSD level, and $\delta E_{int.}^{C-\left(T\right)}$ is the triples correction term to the inter-fragment interaction energy. The electronically excited states were also computed considering the SCS-PBE-QIDH XC functional (Casanova-Páez and Goerigk, 2021) built as the spin-component scaled version of Adamo’s PBE-based double hybrid (Brémond et al., 2014) optimized for excited states, all of them implemented in the same Orca package. Although the TDDFT method can accurately describe the equilibrium geometries of excited states and their energies, it cannot accurately determine the CI points due to the problem of the wrong dimensionality of the CI between ground and excited states at the TDDFT level (Levine et al., 2006; Huix-Rotllant et al., 2016; Huix-Rotllant et al., 2020). Accordingly, in addition to the standard linear-response TDDFT, CI points were also determined by the spin-flip TDDFT (or SF-TDDFT) method (Rinkevicius et al., 2010; Mei and Yang, 2019; Casanova and Krylov, 2020; Matsika, 2021) implemented in the same Orca package. The molecular geometries were built, analyzed, and further manipulated using the Gabedit (Allouche, 2011) and Avogadro (Hanwell et al., 2012), while the molecular graphics were created using the GaussView (Dennington et al., 2009) software.

## 3 Results and Discussions

### 3.1 Benzene–Uracil Model

**FIGURE 1:**
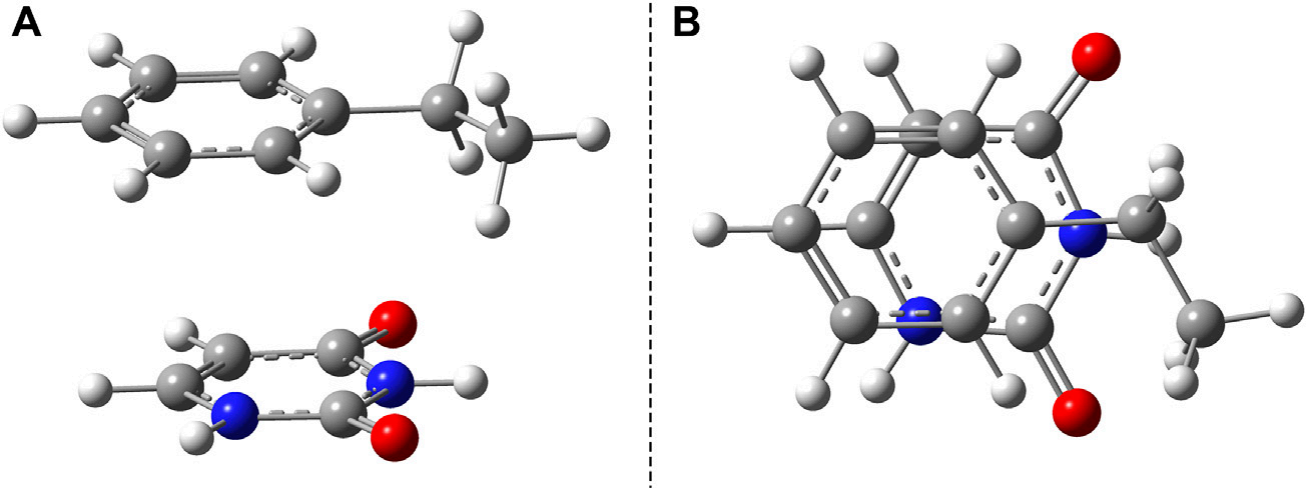
Side **(A)** and top views **(B)** of the uracil–benzene dimer stacking configuration.

The benzene–uracil (B–U) molecular complex can be considered as a model system for describing the light-induced crosslinking reaction between the thymine DNA base (or uracil RNA base) and the phenylalanine-type aromatic side chain of a protein. Accordingly, in the first step, the stacking π– π configuration was built between the aromatic rings of the benzene and uracil. In the case of the benzene ring, the –CH_2_–CH_3_ fragment was also added to mimic the amino acid side chain. Performing geometry optimization without any constrain, using the ωB97X-D3/ma-def2-TZVPP level of theory, the parallel-shifted stacking configuration was obtained. The molecular graphics is presented in Figure 1, where in panel A is shown the side view, while in panel B, the top view of the molecular dimer is given. It is important to note that the stacking configuration of the two aromatic rings is not perfect plane-parallel; the shortest inter-planar distance is 3.435 Å, while the largest one is 3.640 Å. The intermolecular interaction energy between the benzene and uracil units is −10.75 kcal/mol obtained considering the same level of theory used for the geometry optimization. The energy value obtained at the DFT level matches very well with the energy of −11.17 kcal/mol computed considering the DLPNO-CCSD(T) theory. To understand in more detail the nature of the intermolecular interaction, the energy decomposition scheme was applied in the case of DLPNO-CCSD(T) theory. Accordingly, the strength of the interaction without any electron correlation effects is positive (ΔE^*HF*^ = 2.39 kcal/mol), the stacking configuration is stabilized by electron correlation, where the interaction at CCSD (coupled-cluster including singles and doubles cluster operators) is −10.43 kcal/mol, and −11.17 kcal/mol if the perturbative triplet excitation to the cluster expansion is taken into account. The results of the energy decomposition analysis show that the electron correlation effects are mainly covered by the dispersion effects ( $E_{Disp}^{C-CCSD}$ = −12.28 kcal/mol) characteristic for the π– π stacking interaction, which is slightly enhanced by the non-dispersion ( $\delta E_{nonDisp}^{C-CCSD}$ = −0.55 kcal/mol) and the triples correction ( $\delta E_{int.}^{C-\left(T\right)}$ = −0.73 kcal/mol) effects. As can be observed, the dispersion electron correlation effects are decisive in the formation of the stacking configuration, and therefore, theoretical methods that do not take these effects into account cannot correctly describe either the geometry or its electron configuration. Accordingly, to describe electron excitations and their effects on molecular geometry, it is also necessary to use a method that takes these dispersion effects into account.

**FIGURE 2:**
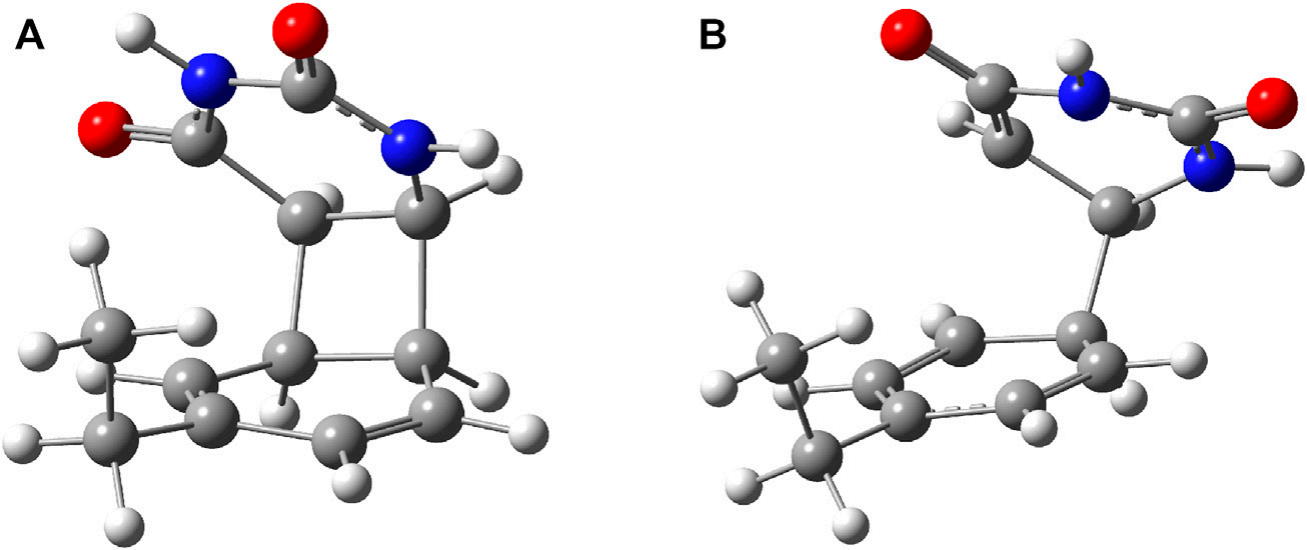
**(A)** The equilibrium geometry of the cyclic adduct and **(B)** the transition state geometry between benzene and uracil units in the benzene–uracil (B–U) complex.

To better understand the structural transition between the stacking-type aggregated complex of the B–U complex and the bounded B–U complex one, not only the “reactants” but also the “product” structure needs to characterize, too. Accordingly, the equilibrium geometry of the CA structure was computed using the same ωB97X-D3/ma-def2-TZVPP level of theory followed by the NEB-type calculation for finding the TS geometry between the stacked and CA geometries. The graphics of the CA and TS geometries are compiled in Figure 2. In the CA configuration case, two saturated bond formations can be observed, through which the aromatic nature of the benzene ring is broken. The C–C bond lengths between the two molecular rings are equivalent, each of them having 1.565 Å of bond distance, while the lengths of corresponding double bonds are modified from 1.390 to 1.552 Å in the case of the benzene and from 1.343 to 1.559 Å for the uracil’s hexagonal ring. In the case of the TS geometry, only a single C–C bond of 1.567 Å length is formed between the two rings, but the aromatic of the benzene and the double-bond character of the uracil rings remains broken as it was found for the CA geometry. The conformation energy (ΔE = E^*stack*^ − E^*CA*^) between the stacking and CA geometries is −29.23 kcal/mol, while the “left” barrier (ΔE = E^*stack*^ − E^*TS*^) between the stacking and TS geometries is −70.33 kcal/mol as well as the “right” barrier [(ΔE = E^*CA*^ − E^*TS*^)] between the CA and TS geometries is −41.10 kcal/mol. Both “left”- and “right”-barrier energy values are given by the TS geometry, indicating that thoroughfare between the two final structures is less likely since the barriers are high enough to keep stable the two equilibrium geometries, and it is even less likely that stacking geometry will flip over in CA geometry. Accordingly, it can be concluded that in the ground state, the stacking B–U complex is not able to change into the CA-type dimerized configuration.

**FIGURE 3:**
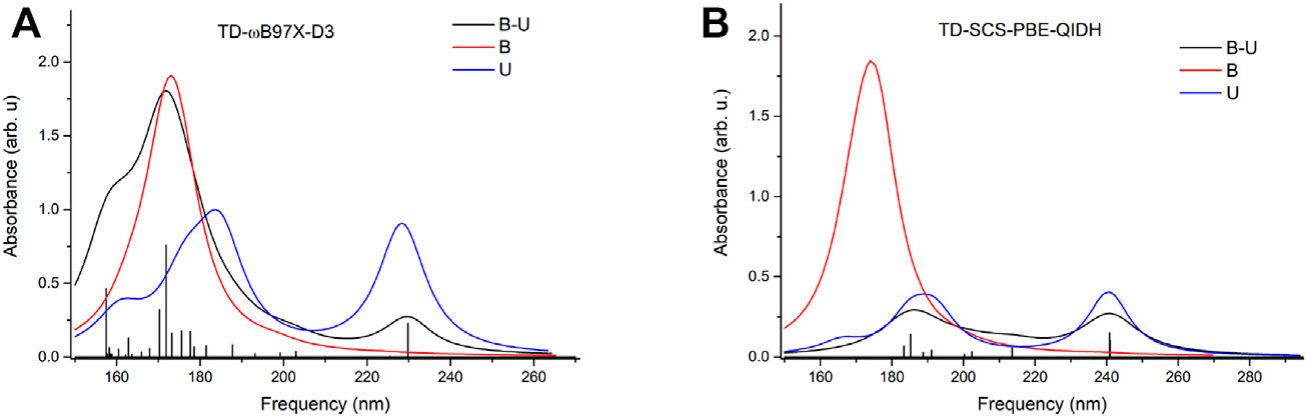
The theoretical UV spectra (black) obtained considering the ωB97X-D3 **(A)** and SCS-PBE-QIDH **(B)** XC functionals. The UV spectra of the individual benzene (red) and uracil (blue) are also included.

**FIGURE 4:**
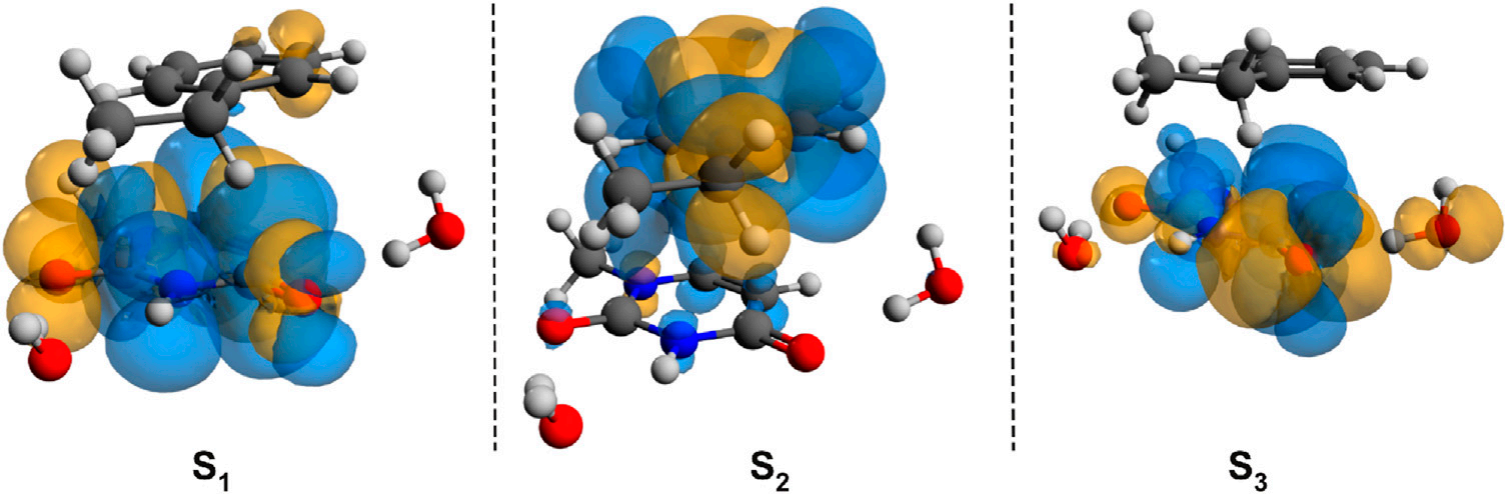
The Natural Difference Orbitals (orange = hole and blue = electron) computed for the first three low-lying excited states of the benzene–uracil (B–U) complex using the TD-ωB97X-D3/ma-def2-TZVPP level of theory.

To check whether this geometry transition can occur at the electronic excited-state level, TDDFT analysis was initiated. Accordingly, the first three, low-lying electronic excited states for the B–U stacking complex computed at TD-ωB97X-D3/ma-def2-TZVPP level of theory are S_1_ = 233 nm, S_2_ = 226 nm, and S_3_ = 218 nm. Since, in the case of ωB97X XC functional, the dispersion effects are not explicitly taken into account in the correlation part of the functional that is only an empirical post-correction to the energy calculation, the double-hybrid SCS-PBE-QIDH XC functional was also considered. The same first three low-lying electronic excited states computed at this time using the TD-SCS-PBE-QIDH/ma-def2-TZVPP level of theory are S_1_ = 241 nm, S_2_ = 226 nm, and S_3_ = 214 nm. The theoretical UV spectra of the B–U complex obtained considering the ωB97X-D3 (left panel) and SCS-PBE-QIDH (right panel) XC functionals are shown in Figure 3, while the Natural Difference Orbitals (NDOs) computed for the first three low-lying excited states of the B–U complex using the TD-ωB97X-D3/ma-def2-TZVPP level of theory are presented in Figure 4. The results presented in Figures 3, 4 show that the S_1_ electronic excited state is mainly localized on the uracil, the S_2_ state on the benzene, and the S_3_ state again on the uracil unit of the B–U complex. It is also important to note that among the three electronic excited states, only the S_1_ state presents significant oscillator strength and accordingly efficient absorption of electromagnetic radiation.

To get a much more comprehensive picture of the absorption of electromagnetic radiation, it is worth comparing the B–U binary system with the isolated uracil and benzene cases themselves. The low-lying excited states of the uracil were investigated in detail by Epifanovsky et al. (2008), showing that in the gas phase the S_0_ → S_1_ excitation is a dark, electronically forbidden *n* → *π* ^⋆^ transition with ω = 237 nm (*f* = 0.0) frequency obtained at EOM-CCSD/aug-ANO-DZ (equation-of-motion coupled-cluster with single and double excitations) level of theory, while the second S_0_ → S_2_ excitation with *π* → *π* ^⋆^ character has a very strong absorption intensity with ω = 222 nm (*f* = 0.2110) frequency. These values are in a very good agreement with the present results obtained at TD-ωB97X-D3/ma-def2-TZVPP level of theory, showing S_1_ = 237 nm (*f* = 0.00003) and S_2_ = 223 nm (*f* = 0.2682) excitation energy values. On the other hand, the inclusion of the solvent (water) affects the excitation energies of the two excited states, where the order of the two excited states, which were already close to each other in the gas phase, is simply reversed, and the energy values are blue-shifted: S_1_ = 228 nm (*f* = 0.3485) and S_2_ = 219 nm (*f* = 0.0001). Regarding the solvent effect, a similar phenomenon can be observed for the binary system B–U. In the gas phase, the two electronic transitions are S_1_ = 233 nm (*f* = 0.00005) and S_2_ = 226 nm (*f* = 0.0616) obtained at TD-ωB97X-D3/ma-def2-TZVPP level of theory, while in water using CPCM solvent model, they are S_1_ = 230 nm (*f* = 0.2295) and S_2_ = 225 nm (*f* = 0.0064), with the difference that it is not the two excited states localized on uracil that is being reversed but one excited state of the uracil with one of the benzene.

**FIGURE 5:**
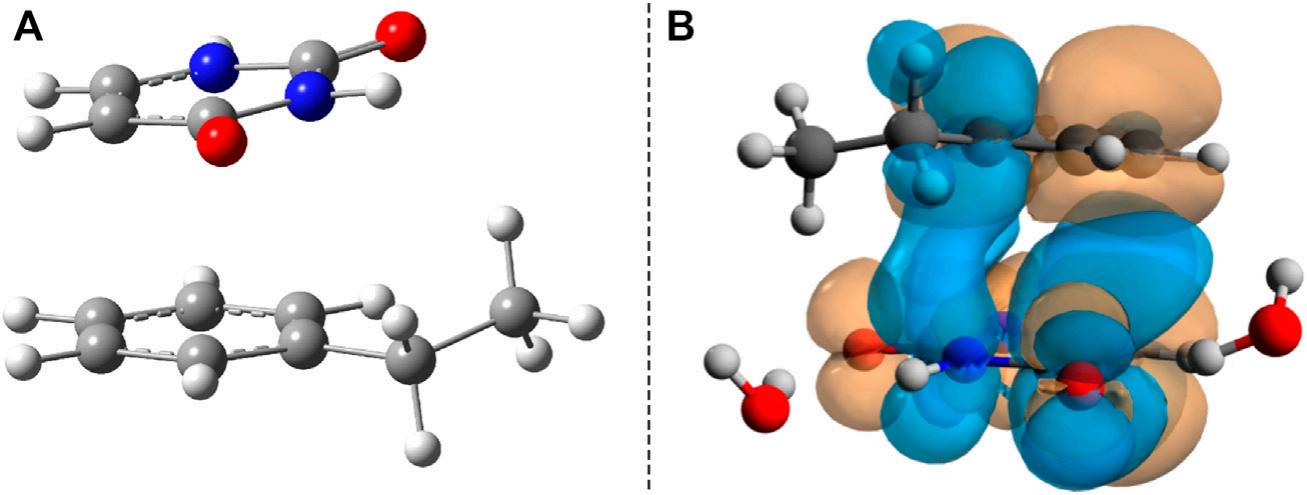
**(A)** The intermediate geometry along the S_1_ energy relaxation channel. **(B)** The Natural Difference Orbitals (NDO) of the S_1_ excited state taken for the intermediate geometry.

**FIGURE 6:**
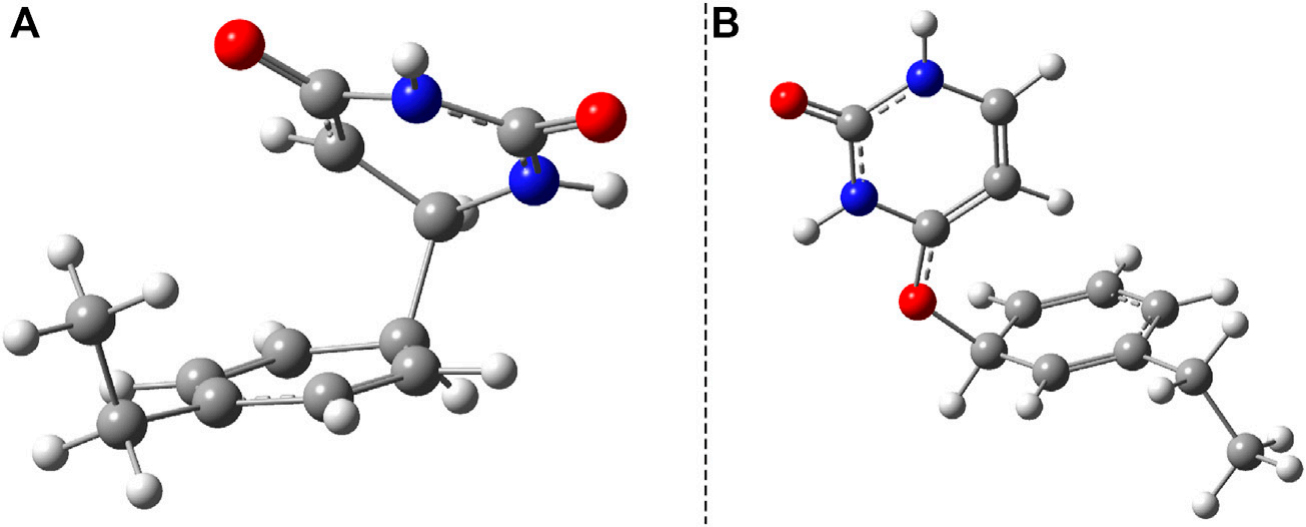
The two (S_0_ ⊗S_1_)_*a*_ and (S_0_ ⊗S_1_)_*b*_ conical intersection geometries found for the benzene–uracil (B–U) binary complex obtained at SF-TD-ωB97X-D3/ma-def2-TZVPP level of theory.

In summary, the analysis of the vertical excitation energies indicates the fact that inside the B–U binary complex, the lowest energy of the electromagnetic radiation is absorbed by the uracil units. The next step would be the analysis of the first electronic excited-state relaxation pathway. Since no real adiabatic dynamics is performed, using a simple energy optimization technique, one can follow only the energetic behavior of the relaxation path. Accordingly, starting the geometry optimization from the ground state geometry configuration and using the same TD-ωB97X-D3/ma-def2-TZVPP level of theory, there are intermediate geometries (not an energetically stable conformation) where the two hexagonal rings get closer to each other and the planar shift of the rings vanishes, as well as the NDO already overlaps the two hexagonal rings. For molecular graphics of one of these geometries, see Figure 5A, while the NDO of its S_0_ → S_1_ transition is presented in Figure 5B. This particular intermediate geometry was chosen only to illustrate how the excited electron charge distribution changes during relaxation. Accordingly, the two rings start to close each other, and the excited electron that was initially localized on the uracil spreads also over the benzene ring, inducing a strong charge transfer. For the chosen intermediate geometry, this charge transfer is 0.24 e, moving from B to U (based on the Löwdin population analysis). Finally, the optimization of the S_1_ excited-state energy falls close to an (S_0_ ⊗S_1_)_*a*_ CI point. In the next step, considering as starting geometry the configuration of the last geometry step during the previous S_1_ excited-state optimization, a searching procedure of the CI was performed. The geometry of the successfully localized CI point is shown in Figure 6A.

**FIGURE 7:**
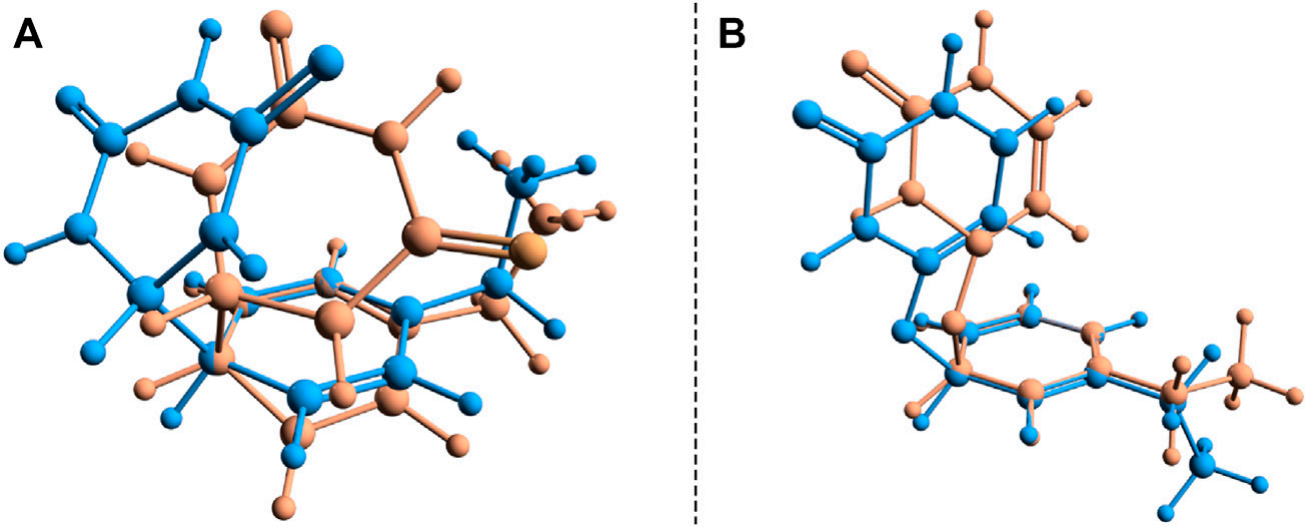
Structural deviation for the two (S_0_ ⊗S_1_)_*a*_ and (S_0_ ⊗S_1_)_*b*_ conical intersection points found for the benzene–uracil (B–U) binary complex computed with the standard (orange) and spin-flip (blue) TDDFT methods.

During the first excited-state geometry optimization, the B–U binary complex arrives at a crossing point between the S_1_ excited and ground states potential energy surfaces, where it loses its excited-state character and turns to the ground-state electron configuration. Performing NEB studies on the nature of the potential energy surface on the one hand between the stacking equilibrium and the S_0_ ⊗S_1_ CI geometries and on the other hand between the CA equilibrium and the S_0_ ⊗S_1_ CI geometries, no TS geometry was found in either case. This means that after the changing of the S_1_ electronic excited state to the S_0_ ground state, the system almost randomly can choose its relaxation path either to the stacking or to the CA geometry. In this way, it can be stated that the excitation considering the first excited state of the stacking configuration of the B–U binary complex finally leads either to back the stacking configuration via the radiationless de-excitation or to the CA configuration via the dimerization reaction. Due to the high enough energy barrier, the CA configuration remains stable, which means that the external electromagnetic radiation to some extent can induce DPC via the CA dimerization. It is well-known that due to the wrong dimensionality of the branching space, the standard TDDFT breaks down near an S_0_⊗S_1_ and fails to correctly compute the CI geometries. On the other hand, it was already proven that dynamic electron correlation counted by dispersion effects is essential to incorrectly describe the supramolecular complex. Therefore, simply applying the multireference HF methods without any perturbational or configuration interaction type correction will also lead to an incorrect description. In this respect, the SF approach of the TDDFT provides a more balanced description of the topology of the branching space. Accordingly, the (S_0_⊗S_1_)_*a*_ geometry was reoptimized considering the SF-TDDFT method. The structural deviation between the standard and the SF versions of the TDDFT is shown in Figure 7A. The major difference can be observed in the orientation of the uracil fragment compared with the benzene unit. More precisely, the new bond formed between the uracil and benzene units changes from the 1.659 Å obtained for the initial case to 1.591 Å for the SF case, as well as rotates the uracil fragment with an additional 20° relative to the benzene ring. This additional rotation means that the C–C bond distance, in which the further decrease is necessary to obtain the CA configuration, will be larger with more than 0.3 Å, and thus, the likelihood of CA configuration also decreases. Furthermore, from the energetic point of view, SP-TDDFT also reduces the height of the Δ(E^*stack*^ − E^*CI*^) energy barrier by ≈20 kcal/mol. Despite the differences, the standard TDDFT can also estimate a near-good S_0_ ⊗S_1_ geometry, but of course, a more accurate method, like SF-TDDFT, is needed to describe them accurately.

Another crossing point between the S_1_ excited and ground state on the potential energy hyper-surface was also found, which, in turn, is unlikely to lead to the formation of a CA configuration. The molecular graphics of this CI crossing point geometry is presented in Figure 6B, where it can be seen that one of the carboxyl groups from the uracil units enlarges its C═O bond, turning to a single bond, and the oxygen makes a new covalent bond with one of the carbon atoms from the benzene ring. The length of the C^*U*^–O bond obtained with the standard TDDFT method is 1.417 Å, while that of the C^*B*^–O bond becomes 1.458 Å. This CI geometry is a very interesting configuration because if one starts a geometric optimization from this position, the system turns to the stacking conformation. This conformational state is located at an energy value of 166.2 kcal/mol higher than the energy of the stacking geometry. The interesting thing about both CI cases is that not only the uracil or benzene ring is formed, but in both cases, the joint deformation of the two rings can be observed. As seen earlier, in addition to the standard TDDFT, another more advanced method is needed to accurately describe the CI geometry. Accordingly, the (S_0_⊗S_1_)_*b*_ geometry was again reoptimized, considering the SF-TDDFT method. The structural deviation between the standard and the SF versions of the TDDFT is shown in Figure 7B. The results show that the length of the C^*U*^–O bond decreases to 1.333 Å, and that of the C^*B*^–O bond increases to 1.524 Å compared with the initial geometry parameters, while the corresponding energy barrier decreases with ≈57 kcal/mol.

**FIGURE 8:**
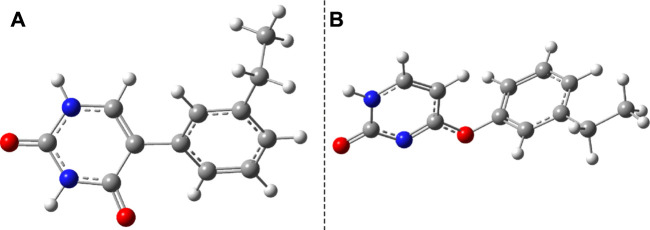
Two possible photoproduct geometries with direct covalent bond for the benzene–uracil (B–U) binary complex: **(A)** C–C bridge and **(B)** O–H bridge.

Another important aspect is what other kinds of photoproduct geometries can be formed after the irradiation and how energetically they seem to be real. Accordingly, new binary complexes considering different direct bonding possibilities were built, and their geometries were optimized. In the first complex, the uracil and benzene rings are bounded through the C–C single covalent bond (Figure 8A), while in the second case, the O atom of the uracil makes a covalent bond with a C atom from the benzene ring (Figure 8B). It is important to note that in both cases, the formation of photoproducts takes place through several reaction steps. While in the first case we are talking about a two-step process where, in addition to the formation of the covalent bond, dehydrogenation also takes place, in the second case, an intermediate step of a bond conjugation also happens. If one compares these geometries energetically to the stacking configuration, it can be found that the conformational energy difference for case *a*) is +19.17 kcal/mol (E^*diff*^ = +29.23 kcal/mol) while for case *b*) is +45.98 kcal/mol. The geometry found in the first case seems to be more stable even compared with the CA configuration, but it is a question of what successive energy barriers need to be overcome for the reaction to take place. One possible turning point is the formation of the (S_0_⊗S_1_)_*b*_ CI point (Figure 6B) since in this case the aromatic nature of the benzene ring is already broken, and it may be possible for dehydrogenation to take place.

### 3.2 Phenol–Uracil Model

**FIGURE 9:**
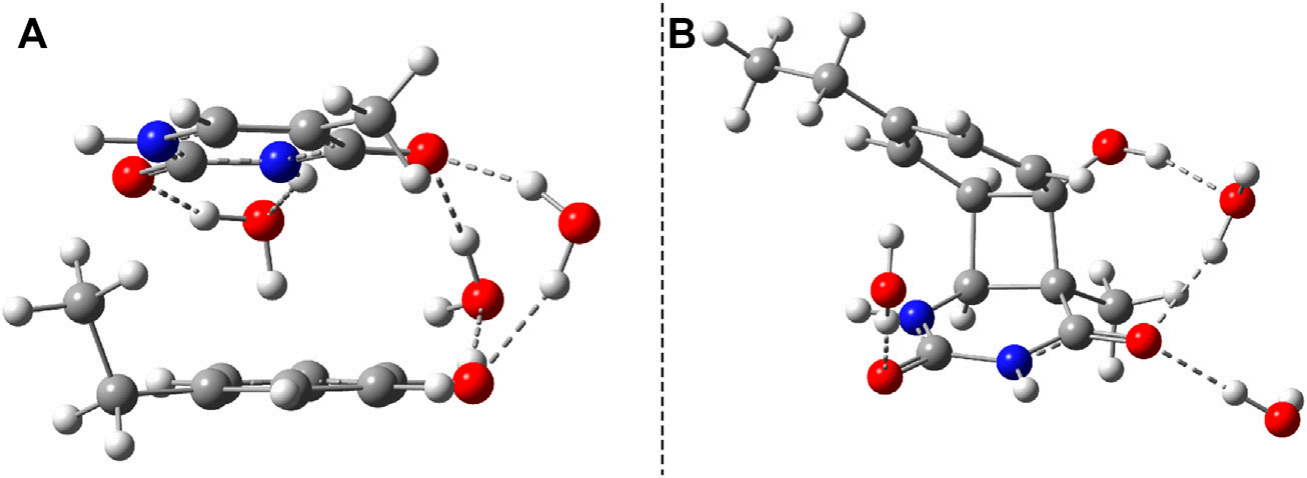
The equilibrium geometries of the stacking **(A)** and cyclic adduct **(B)** between phenol and uracil units in the phenol–uracil (P–U) complex obtained at the TD-ωB97X-D3/ma-def2-TZVPP level of theory.

Considering the phenol–uracil (P–U) model system, one can model the light-induced crosslinking reaction for the phenylalanine–uracil protein–DNA cases. The geometry optimization of the P–U binary complex results in a quasi-stacking parallel configuration where the tilted position is induced by the hydrogen-bond network made by the three water, the O atom of the uracil, and the OH fragment of the phenol units (Figure 9A). In this case, the water molecules were also depicted in the molecular graphics. The shortest inter-planar distance (O…O) is 3.490 Å, while the largest one is 3.870 Å. The intermolecular interaction energy obtained at the DFT level of theory is −7.87 kcal/mol, while that found considering the DLPNO-CCSD(T) theory is −8.47 kcal/mol. The stacking configuration is exclusively stabilized by the dispersion-type electron correlation effects, in which their contributions to the final intermolecular binding energy are −8.89 kcal/mol.

**FIGURE 10:**
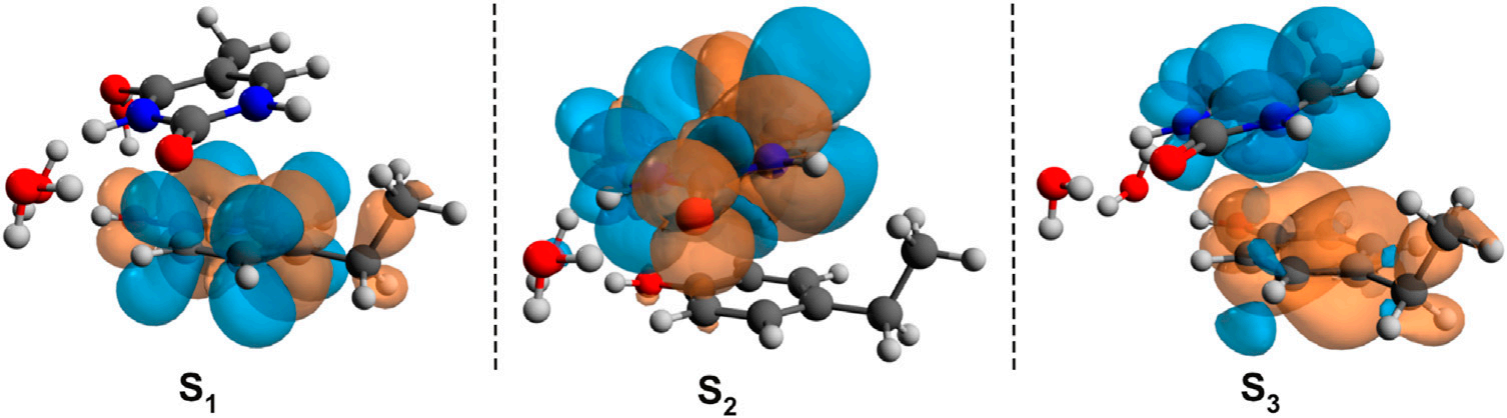
The Natural Difference Orbitals (orange = hole and blue = electron) computed for the first three low-lying excited states of the phenol–uracil (P–U) complex using the TD-ωB97X-D3/ma-def2-TZVPP level of theory.

The first low-lying electronic excited states were computed using the ωB97X-D3 and SCS-PBE-QIDH XC functionals. The excitation energies are as follows: S_1_ = 238 nm, S_2_ = 231 nm, and S_3_ = 223 nm in the case of ωB97X-D3 and S_1_ = 254 nm, S_2_ = 240 nm, and S_3_ = 226 nm for the SCS-PBE-QIDH double-hybrid XC functional. The most significant difference from the previously studied system is that in the present case the first excited state is localized on the phenol unit and not on the uracil component as one was found for the B–U case. The second electronic excited state is almost entirely uracil in nature, while the third one is a P → U charge transfer state. For their NDOs, see Figure 10.

**FIGURE 11:**
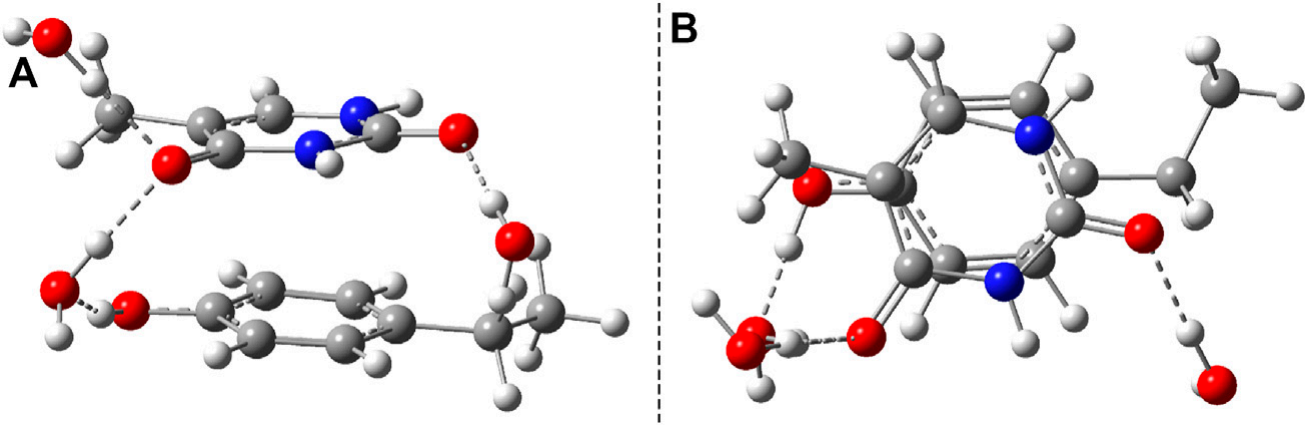
Side **(A)** and top views **(B)** of the S_1_ excited-state equilibrium geometry configuration of phenol–uracil (P–U).

After the calculation of the vertical excitation energies, the equilibrium geometry of the excited state S_1_ was also determined. The molecular graphics of the S_1_ equilibrium geometry is shown in Figure 11. The geometric changes compared with the ground state geometry are manifested in the fact that the two hexagon rings get closer (the closest interatomic distance is 2.927 Å) and rotate in the plane at 60° relative to each other. It is characteristic of both the P–U and B–U systems that the distance between the stacking planes decreases during the S_1_ relaxation. From this point of view, it may even seem irrelevant on which fragment the vertical excitation is located.

Similar to the B–U system, the CA-type equilibrium geometry of the P–U binary complex was also computed, and its geometry is shown in Figure 9B. Unlike the B–U system, in the P–U binary complex case, the C–C vertical bond lengths between the two molecular rings are not equivalent (1.562 and 1.587 Å); the carbon atom of the benzene ring that binds also the OH fragment makes a bit larger C–C vertical bond than the other C–C bond. The conformational energy is 34.53 kcal/mol higher compared with the stacking system. With the use of the NEB TS geometry searching method, the TS geometry between the stacking and CA configurations was also computed. The “left” barrier (ΔE = E^*stack*^ − E^*TS*^) between the stacking and TS geometries is −44.95 kcal/mol as well as the “right” barrier (ΔE = E^*CA*^ − E^*TS*^) between the CA and TS geometries is only −10.43 kcal/mol. This latter value means that the CA geometry is no longer as stable as it was found for the B–U system. In this way, the dimerized conformation (CA) and implicitly the crosslinked P–U configuration are less probable to be formed, and thus during a possible CI-driven relaxation, the probability of CA geometry appearing is lower. Of course, all these findings are true when the carbon atom to which the phenyl group is bound takes part in the CA formation. When this is not the case, namely, the CA is formed between two benzene-like carbon atoms, then, of course, the configuration already analyzed for the B–U system will be valid with a good approximation.

**FIGURE 12:**
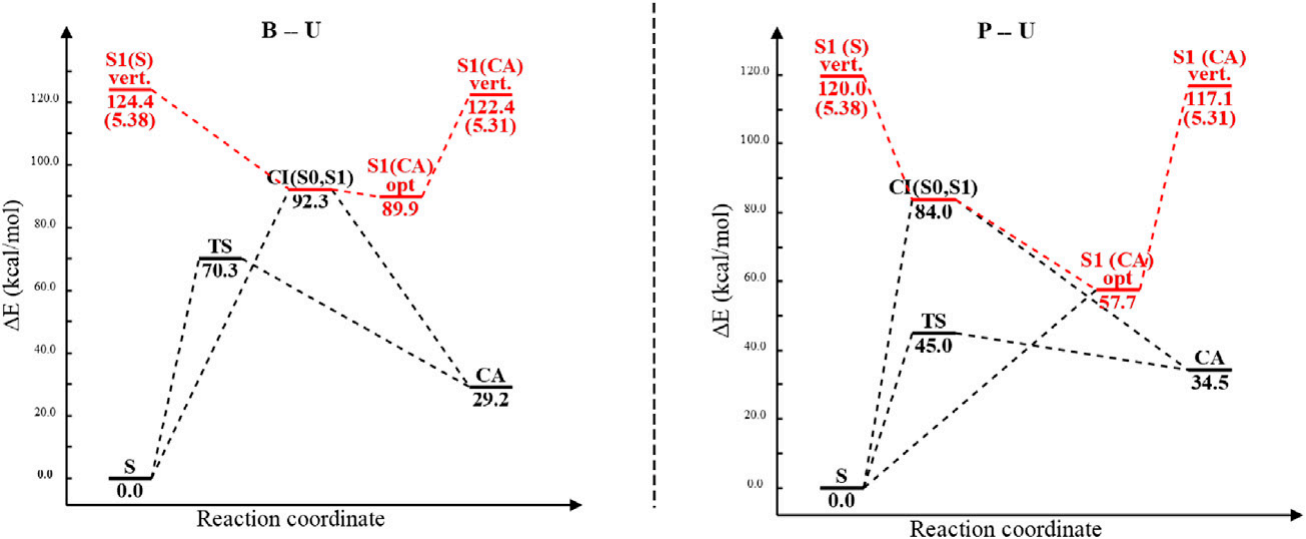
The potential energy profiles including equilibrium geometry, vertical excitation, transition state, and conical intersection point energies for the benzene–uracil (B–U) and phenol–uracil (P–U) complexes. Energy values of different conformational barriers are given in kcal/mol, while those in parentheses are in eV. Red path color means the electronic excited-state relaxation, while the blue one represents the ground electronic state relaxation.

Analyzing the potential energy profiles both for the B–U and for the P–U cases shown in Figure 12, it can be said that relaxation after vertical excitation of either stacking or CA structures very easily leads to the (S_0_⊗S_1_)_*a*_ CI geometry and from there returns to the ground state electron structure of one of the structures without spending much time in their excited state.

## 4 Conclusion

In the present work, considering the B–U and phenol–uracil model systems, the light-induced DPC reaction was investigated based on the ωB97X-D3 and SCS-PBE-QIDH XC DFT functionals as well as DLPNO-CCSD (T) coupled-cluster theory. The main focus was oriented on the possible occurrence of CA reactions similar to thymine dimerization in DNA (Mendieta-Moreno et al., 2016). The result obtained for the first excited-state relaxation pathway of B–U shows that the S_1_ state presents a spontaneous relaxation till it reaches the supramolecular-type (S_0_⊗S_1_)_*a*_ CI point, defined by the deformation of both uracil and benzene moieties, from which the B–U binary system slides almost randomly into either stacking or CA-type geometry. From this, it can be concluded that during the photo-reaction induced by the external electric field, both biologically positive internal conversion, serving as a protection mechanism against the UV radiation, and biologically negative dimerization can occur as possible scenarios. In the case of the phenol–uracil binary system, where the carbon atom that holds the OH group is involved in the CA-type binding, the occurrence of the CA product is less possible, whereas the energy barrier between the TS and the CA-type geometries is much lower than that found for the B–U system. This can certainly be explained by the presence of the OH group in the phenol unit. At the same time, we also found another supramolecular-type CI configuration (S_0_⊗S_1_)_*b*_, which in our view could be the starting point of the multistep reaction during which crosslinking configurations known in the literature (Spitzer et al., 2014; Hafner et al., 2021) are formed.

## Data Availability

The original contributions presented in the study are included in the article/Supplementary Material. Further inquiries can be directed to the corresponding author.
